# Transcriptome, Methylome and Genomic Variations Analysis of Ectopic Thyroid Glands

**DOI:** 10.1371/journal.pone.0013420

**Published:** 2010-10-15

**Authors:** Rasha Abu-Khudir, Jean Paquette, Anne Lefort, Frederick Libert, Jean-Pierre Chanoine, Gilbert Vassart, Johnny Deladoëy

**Affiliations:** 1 Department of Pediatrics, Endocrinology Service and Research Center, CHU Sainte-Justine, University of Montreal, Montreal, Canada; 2 Department of Biochemistry, University of Montreal, Montreal, Canada; 3 IRIBHM, Free University of Brussels ULB, Brussels, Belgium; 4 Endocrinology and Diabetes Unit, Department of Pediatrics, BC Children's Hospital, University of British Columbia, Vancouver, Canada; Ohio State University Medical Center, United States of America

## Abstract

**Background:**

Congenital hypothyroidism from thyroid dysgenesis (CHTD) is predominantly a sporadic disease characterized by defects in the differentiation, migration or growth of thyroid tissue. Of these defects, incomplete migration resulting in ectopic thyroid tissue is the most common (up to 80%). Germinal mutations in the thyroid-related transcription factors NKX2.1, FOXE1, PAX-8, and NKX2.5 have been identified in only 3% of patients with sporadic CHTD. Moreover, a survey of monozygotic twins yielded a discordance rate of 92%, suggesting that somatic events, genetic or epigenetic, probably play an important role in the etiology of CHTD.

**Methodology/Principal Findings:**

To assess the role of somatic genetic or epigenetic processes in CHTD, we analyzed gene expression, genome-wide methylation, and structural genome variations in normal *versus* ectopic thyroid tissue. In total, 1011 genes were more than two-fold induced or repressed. Expression array was validated by quantitative real-time RT-PCR for 100 genes. After correction for differences in thyroid activation state, 19 genes were exclusively associated with thyroid ectopy, among which genes involved in embryonic development (e.g. *TXNIP*) and in the Wnt pathway (e.g. *SFRP2* and *FRZB*) were observed. None of the thyroid related transcription factors (FOXE1, HHEX, NKX2.1, NKX2.5) showed decreased expression, whereas PAX8 expression was associated with thyroid activation state. Finally, the expression profile was independent of promoter and CpG island methylation and of structural genome variations.

**Conclusions/Significance:**

This is the first integrative molecular analysis of ectopic thyroid tissue. Ectopic thyroids show a differential gene expression compared to that of normal thyroids, although molecular basis could not be defined. Replication of this pilot study on a larger cohort could lead to unraveling the elusive cause of defective thyroid migration during embryogenesis.

## Introduction

Permanent primary hypothyroidism is the most common congenital endocrine disorder. In up to 85% of cases, it results from thyroid dysgenesis, a condition comprised of defects in the differentiation, migration or growth of thyroid tissue. Of these defects, incomplete migration resulting in ectopic thyroid tissue (sub-lingual thyroid) is the most common (up to 80%). The etiological diagnosis is established through thyroid scintigraphy [1]. Ectopic thyroids are smaller (i.e. they lack the lateral lobes that are characteristic of orthotopic thryroids) but are otherwise normal (i.e. they have a normal follicular architecture and their capacity to trap and organify iodine and to produce thyroid hormones and thyroglobulin is intrinsically normal [2], [3], [4], [5]). This suggests that the hypothyroidism of subjects with thyroid ectopy is due to a smaller amount of tissue (hypoplasia), which is a consequence of the migration defect, and not to defects in differentiation or in histological organization of the thyroid follicular cells.

Congenital hypothyroidism from thyroid dysgenesis (CHTD) is a heterogeneous disease, which exists in familial (2%) and non-familial (sporadic, 98%) forms [6]. Moreover, the results of a survey of monozygotic twins yielded a discordance rate of 92% [7], which together with the female predominance in CHTD [8] suggest that complex non-Mendelian mechanisms underlie this condition. On the other hand, environmental causes operating *in utero* are unlikely because: (i) no temporal or seasonal trends for CHTD have been observed [9] and (ii) MZ twins who are discordant for CHTD have similar birth weight (G. Van Vliet, personal communication). Germinal mutations in thyroid related transcription factors NKX2.1, FOXE1, PAX-8, and NKX2.5 have been identified in only 3% of patients with sporadic CHTD and linkage analysis excluded these genes in some multiplex families with CHTD, which is consistent with a complex genetic contribution [10]. Together these findings indicate the involvement of novel genes and pathways and underlines the importance of somatic epigenetic or genetic events [11].

**Figure 1:**
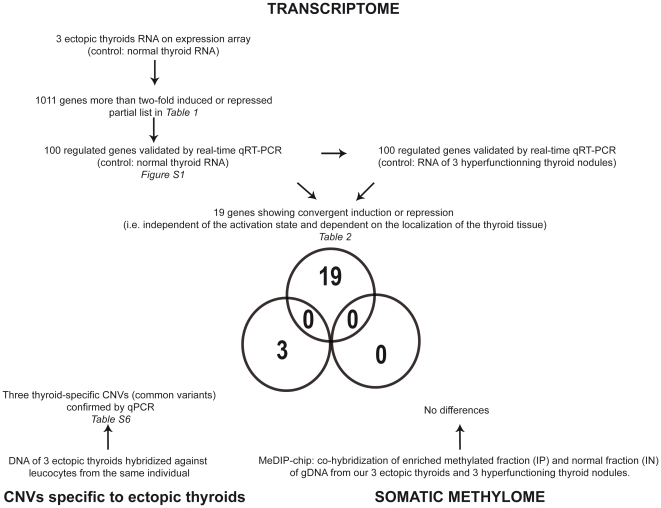
Breakdown of the experimental design and overview of the results.

Combining data of gene expression, DNA methylation and DNA copy number has led to the identification of novel genetic regulators of cancer [12], [13]. Consistent with this approach, we aim to assess whether the transcriptome of ectopic thyroids is shaped by somatic genomic or epigenomic variations (Figure 1).

## Results

### Expression array identified 1011 genes that are more than two-fold induced or repressed

We used microarray analysis to compare the genome-wide RNA expression profile of normal (orthotopic; n = 1) versus abnormal (ectopic; n = 3) thyroid tissue. We identified 1833 differentially expressed genes, and a total of 1011 genes were induced (n = 522) or repressed (n = 489) more than two-fold. The forty genes with the highest differential expression are listed in Table 1. To validate the differential expression identified by microarray analysis, we performed quantitative real-time PCR (qRTPCR) of 100 genes in ectopic thyroids compared with the same commercial control (Ambion) used for the arrays; these 100 genes included highly differentially expressed genes and genes known to play a role in the thyroid function. Overall, there was a highly significant correlation between microarray and qRTPCR (Pearson correlation coefficient of 0,86, p<2.2 e-16)(Figure S1).

**Table 1:** Result of the expression array: the top twenty induced (upper panel) and top twenty repressed (lower panel) genes.

TOP 20 induced genes				
Entrez Gene Name	Entrez Gene ID	Array^a^	q-value (%)^b^	qRTPCR RQ value^c^
**LYZ**	4069	5,99	0,0000	5,41
**FOSB**	2354	5,48	0,0000	5,62
IGJ	3512	5,44	0,0000	
TRA@	6955	5,23	0,7897	
**PAX8**	7849	4,68	0,0000	2,55
CYBB	1536	4,62	0,0000	
TRA@	6955	4,60	0,0000	
HLA-DQA1	3117	4,56	0,0000	
CECR1	51816	4,49	0,0000	
HLA-DQB1	3119	4,41	0,0000	
EGR1	1958	4,29	0,0000	
RNASE6	6039	3,98	0,0000	
**SFRP2**	6423	3,91	0,0000	4,74
**GPNMB**	10457	3,87	0,0000	4,97
PABPC1	26986	3,85	0,0000	
MS4A6A	64231	3,84	0,0000	
KLF4	9314	3,74	0,7897	
HADHA	3030	3,61	0,7897	
**FOS**	2353	3,61	0,0000	4,46
IGHG4	3503	3,52	0,7897	

TOP 20 repressed genes				
Entrez Gene Name	Entrez Gene ID	Array^a^	q-value (%)^b^	qRTPCR RQ value^c^
**ABCA13**	154664	−6,06	0,0000	−4,12
**CKM**	1158	−5,86	0,0000	−10,31
**ACTA1**	58	−5,31	0,0000	−5,06
C9orf70	84850	−4,85	0,0000	
**CHGA**	1113	−4,83	0,0000	−8,38
MYBPC1	4604	−4,60	0,0000	
ATP2A1	487	−4,19	0,0000	
**TNNC2**	7125	−4,09	0,0000	−4,64
IGSF1	3547	−4,00	0,0000	
EEF1A2	1917	−3,87	1,9070	
FLJ32115	121506	−3,79	0,7897	
PCSK2	5126	−3,66	1,9070	
**RASD1**	51655	−3,53	2,7615	−0,51
RARRES1	5918	−3,52	1,9070	
**EDN3**	1908	−3,40	1,9070	−1,83
PRKCE	5581	−3,32	2,7615	1,98
GSTT1	2952	−3,24	4,2100	
FLJ11127	54491	−3,20	3,2882	
TCAP	8557	−3,17	3,9587	
LOC440696	3322	−3,17	3,2882	

a Array mean ratio are expressed in log_2_.

b Q-values (i.e. minimal false discovery rate) are expressed in percent, all P-values are less than 0.001.

c Validation with qRTPCR: RQ values are expressed in log_2_.

Bold characters indicate genes that are validated (13 of 14 tested) among these 40 genes.

### Functional annotation clustering of the 1011 differentially expressed genes showed enrichment for developmental processes

**Figure 2:**
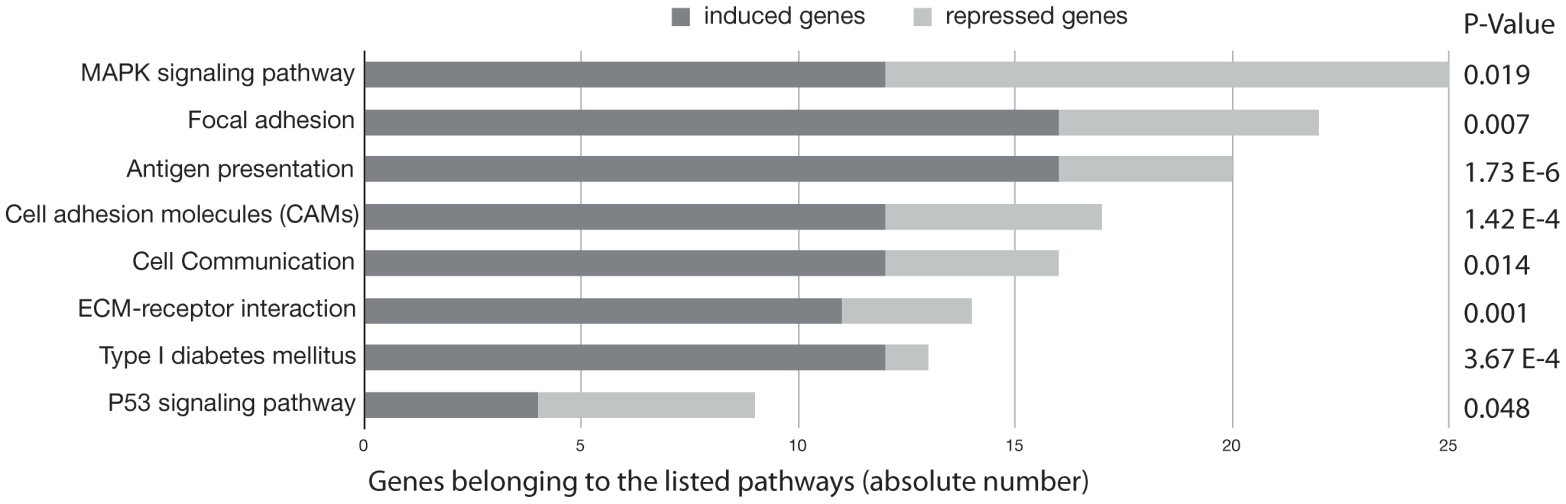
Expression array: gene pathway distribution in ectopic thyroid according to KEGG. The pathways are classified according to the number of regulated genes (HEEBO array results). P-values of Fisher's exact test for each KEGG pathways are listed to the right of the boxes. *Dark gray*, induced genes; *gray*, repressed genes.

To assess whether the differentially expressed genes are related to development and organogenesis, we classified the 1011 differentially expressed genes into gene ontology (GO) groups using DAVID (Database for Annotation, Visualization and Integrated Discovery) with medium classification stringency. Table S1 shows the top three clusters for the 1011 differentially expressed genes (more than two-fold induced or repressed) with enrichment scores greater than 6 (i.e. p<1 E -06). Two of them are clusters of genes enriched for developmental processes. We next clusterized separately the induced (n = 522) and the repressed (n = 489) genes using DAVID according to GO terms with high classification stringency. The top five clusters of induced genes with enrichment scores greater than 5.5 (i.e. p<0.5 E -06) show genes important for development, vasculogenesis, the extracellular matrix, immune system development and collagen whereas the top five clusters for repressed genes with enrichment scores greater than 4 (i.e. p<1 E -04) show genes important for histone function, apoptosis, chromatin function, organelle and contractile functions (Table S2). Finally, the analysis of Kyoto Encyclopedia of Genes and Genomes (KEGG) pathways with DAVID shows enrichment (p<0.05) for eight pathways, among which three are associated with cell-to-cell interaction (i.e. MAPK signaling pathway, focal adhesion and cell communication) (Figure 2).

### Additional validation against age-matched hyperfunctioning thyroid tissue selects 19 genes whose expression is related to thyroid ectopy and independent of thyroid activation state

Level of activity (i.e. thyroid hormone production) and consequently expression of genes involved in thyroid hormone production is sensitive to thyroid stimulating hormone (TSH) [14], [15]. CHTD patients have high TSH at diagnosis, after which the level of their TSH may vary depending on the compliance to the treatment; or the TSH is high for years in cases of delayed treatment, as in our case 1 (Table S3). To correct for differential TSH-dependent activation of thyroid tissue, we extended the qRTPCR analysis for the 100 validated genes to three hyperfunctioning thyroid nodules (i.e. somatic activation of the TSH receptor) that had arisen in otherwise normal (orthotopic) thyroids. Of the 100 genes, 19 showed consistently induction or repression when compared to all types of controls (i.e. hyperfunctioning thyroid nodules and the commercial control) (Table 1). The 81 remaining genes showed divergent expression: i.e. they were either induced in ectopic thyroid when compared to the commercial control but repressed when compared to the three hyperfunctioning thyroid nodules or *vice versa* (Table S4), suggesting that the expression of those 81 genes was dependent of thyroid activation state.

### Pathway analysis of the 19 selected genes showed association with Wnt signalling pathway whereas the 81 excluded genes were enriched for thyroid hormonogenesis and function

We then asked whether the use of two different types of control was able to exclude genes that are dependent on thyroid activation state and to identify genes associated exclusively with defective migration of the thyroid. To consider the level of expression (which is not possible with DAVID), we used the Ingenuity Pathways Analysis software for GO annotation and pathway analysis (Ingenuity® Systems, www.ingenuity.com). The 19 selected genes (Table 2) were enriched for the Wnt pathway, dendritic cell maturation pathway, and embryonic developmental functions (for each enrichment scores greater than 1.5; p<0.02 and Benjamini-Hochberg multiple correction p<0.05). As expected, the 81 genes (Table S4) excluded because of divergent expression are enriched for thyroid hormonogenesis and function: thyroglobulin *(TG)*, thyroid peroxidase *(TPO)*, deiodinase type II *(DIO2)*, deiodinase type I *(DIO1)*, dual oxidase 2 *(DUOX2)*, paired box gene 8 *(PAX8)*, thyroid stimulating hormone receptor *(TSHR)* and thyroid hormone responsive SPOT14 homolog *(THRSP)* (i.e for GO term endocrine system disorders, the enrichment score is 2; p<0.01 and Benjamini-Hochberg multiple correction p<0.01). To control for tissue quality, we also analyzed 10 unregulated genes which are either well described thyroid-related transcription factors (FOXE1, HHEX, NKX2.1, NKX2.5) [16] or genes involved in the Wnt pathway (CTNNB1, GSK3B, CDH1, APC, AXIN1, AXIN2) [17]. CTNNB1 and CDH1 showed divergent expression; APC, AXIN1, AXIN2, FOXE1 and NKX2.1 showed increases of convergent expression; and NKX2.5 expression was massively increased when compared to orthotopic thyroids but only slightly increased when compared to the commercial control, which therefore might be considered as divergent. None of the thyroid related transcription factors (FOXE1, HHEX, NKX2.1, NKX2.5) showed decreased expression regardless of the control used (Table 3).

**Table 2:** Validated genes (n = 19) with convergent induced (n = 16) and repressed (n = 3) expression in ectopic thyroid tissue (i.e. independent of the activation state and dependent on the localization of the thyroid tissue).

Entrez Gene Name	Enterz Gene ID	Array^a^	qRTPCR vs normal thyroid^a^	qRTPCR vs hot nodules^a^	Taq Man Assay ID for qRTPCR
**SFRP2**	**6423**	**3,91**	**4,74**	**5,48**	Hs00293258_m1
TBX1	6899	1,52	3,21	1,57	Hs00271949_m1
GPNMB	10457	3,87	4,97	1,45	Hs01095669_m1
DUSP6	1848	1,98	3,09	1,32	Hs01044001_m1
**FRZB**	**2487**	**0,83**	**1,83**	**1,24**	Hs00173503_m1
COL1A1	1277	2,71	5,93	1,10	Hs00164004_m1
FGL2	10875	2,46	4,22	1,00	Hs00173847_m1
LYZ	4069	5,99	5,41	0,94	Hs00426231_m1
COL3A1	1281	3,39	5,12	0,89	Hs00164103_m1
TYROBP	7305	2,25	3,62	0,76	Hs00182426_m1
TNFAIP2	7127	3,45	4,63	0,68	Hs00196800_m1
BGN	633	1,09	3,14	0,63	Hs00959143_m1
PBX4	80714	1,83	3,67	0,58	Hs00257935_m1
PLCXD1	55344	1,71	2,83	0,58	Hs00383111_m1
CPEB4	**80315**	1,28	2,6	0,54	Hs00270923_s1
*MKRN1*	*23608*	*2,63*	*1,18*	*0,53*	Hs00831972_s1
TXNIP	10628	−2,81	−1,78	−0,51	Hs01006900_g1
ABCA13	154664	−6,06	−4,06	−0,71	Hs00541549_m1
ENO3	2027	−1,99	−0,86	−1,15	Hs00266551_m1

a Array mean ratio and qRTPCR RQ are expressed in log_2_.

*Bold* characters indicate genes that are directly associated with the Wnt pathways (canonical and non-canonical).

*Italic* characters indicate genes that are regulators of the Wnt/beta-Catenin pathway [32].

**Table 3:** Quantitative RTPCR for 10 controls.

Entrez Gene Name	Entrez Gene ID	RQ of qRTPCR vs normal thyroid^a^	RQ of qRTPCR vs hot nodules^a^	Taq Man Assay ID for qRTPCR
APC	324	2,31	0,13	Hs01568269_m1
AXIN1	8312	1,39	0,2	Hs00394718_m1
AXIN2	8313	2,05	0,62	Hs01063168_m1
CTNNB1	1499	1,35	−0,53	Hs00355045_m1
CDH1	999	2,07	−0,35	Hs00170423_m1
FOXE1	2304	2,58	0,47	Hs00538731_s1
GSK3B	2932	2,1	0	Hs00275656_m1
HHEX	3087	1,62	0,05	Hs00242160_m1
NKX2.1	16002	1,43	0,36	Hs00163037_m1
NKX2.5	1482	0,53	6,93	Hs00231763_m1

a Array mean ratio and qRTPCR RQ are expressed in log_2_.

### Differential gene expression in ectopic thyroid is independent of methylation

The next step was to assess whether somatic changes in DNA methylation play a role in dysregulation of gene expression in ectopic thyroids. Methylation profiling by methylated DNA immunoprecipitation (MeDIP) and MeDIP-chip was performed by hybridizing pairs of enriched methylated fraction (IP) and normal fraction (IN) of genomic DNA from our three ectopic thyroids and three controls orthotopic hyperfunctioning thyroid nodules. The methylation profile was similar between the ectopic and orthotopic thyroids: after multiple test correction, there was no statistically significant difference (i.e. no region with a less stringent False Discovery Rate threshold of 0.1) (data not shown). Consequently, no correlation was found between the differential expression in ectopic thyroids and the global methylation profile.

### Differential gene expression in ectopic thyroid is independent of thyroid-specific CNVs

To assess whether thyroid-specific (i.e. absent in matched leucocytes) CNVs shape gene expression in ectopic thyroids, we used array comparative genomic hybridization (aCGH) of the ectopic thyroid DNA with matched leucocytes. By analyzing data as described in *Materials and* *Methods*, we found four thyroid-specific CNVs (three validated by qPCR), which are reported variants as reported in the Database for Genomic Variants (http://projects.tcga.ca/variation) (Table S5). No correlation was found between thyroid-specific CNVs and differentially expressed genes in ectopic thyroids.

## Discussion

Generally, CHTD is sporadic and shows discordance between MZ twins [7]. Somatic genetic or epigenetic events might therefore have a role in the etiology of this condition. The objective of this study was to assess whether somatic molecular events account for the failure of migration of ectopic thyroids. Therefore, we conducted the first integrative analysis of transcriptome, DNA methylation and structural variants (CNV) in ectopic thyroids.

We found altered expression in genes and pathways that might play a significant role in abnormal thyroid development (e.g. Wnt signaling pathway). Interestingly, none of the thyroid related transcription factors (FOXE1, HHEX, NKX2.1, NKX2.5) showed decreased expression, whereas PAX8 expression was associated with thyroid activation state. This is a direct indication that the expression of these known candidate genes is at least neutral in ectopic thyroid and is consistent with the observation that the coding sequences for FOXE1, NKX2.1 and PAX8 were normal in case #1 [5].

Four pathways identified by analysing the results of expression arrays (i.e. focal adhesion, antigen processing and presentation, cell communication, cell adhesion molecules and Type I diabetes) have been identified independently in hyperfunctioning thyroid nodules [18]. However, our results identify mostly induced genes in these pathways (Figure 1) whereas repression of these genes was observed in the aforementioned study [18]. To obtain, in ectopic thyroid, an opposite expression pattern when compared to that of orthotopic hyperfunctional thyroid (i.e. thyroid with somatic TSH receptor activating mutation) is plausible, but it underlines also the need to consider the differential activation of the TSH-receptor signaling pathways in our samples. Consequently, we have excluded 81 validated genes for which expression was associated with TSH-driven thyroidal activity.

The 19 selected genes whose expression was dependent on thyroid location (i.e. ectopy) were enriched for pathways involved in cellular movement (i.e. Wnt pathway and dendritic cell maturation pathway). This association has biological plausibility especially for the Wnt pathway. First, non-canonical Wnt pathway is crucial for cell migration [19] and development of organs of endodermal origin (e.g. intestine, lung, pancreas) [20]. There is indirect evidence for the involvement of the non-canonical Wnt pathway in the developing thyroid in mice [21], even though the canonical Wnt/beta-catenin pathway seems to be inactive during thyroid development in mice and humans [21], [22]. Second, as Wnt signaling is implicated in development and cancer [23], to find an association between Wnt pathway and thyroid ectopy (i.e. failure of proper thyroid migration during development) makes biologically sense. Indeed, SFRPs have been associated with embryonic patterning [24], inhibition of meduloblastoma cell proliferation [25] and inhibition of glioma cell motility [26]. Inhibition of the Wnt pathway by Wnt5-a has also been shown to supress tumor activity in thyroid carcinoma [27].

This study has several limitations. First, the expression profiles in tissue collected and analyzed postnatally may not reflect embryonic expression. Consequently, whether the differences we observed are causes or consequences of the ectopic location of the thyroid remains to be tested. Second, even though clusters of genes involved in histone and chromatin function have repressed expression in ectopic thyroids, we have not formally excluded a role of differential histone methylation or acetylation on differential gene expression in ectopic thyroids. Third, the arrays used for the CNVs and methylome analyses have their own limits in definition and genome coverage. Lastly, the sample number is small but our preliminary findings justify testing a larger number of samples.

This study identifies interesting candidate pathways that may play important roles in the migration of the embryonic thyroid and provides a prototype approach for the study of congenital disorders difficult to explain by classical genetics.

## Methods

### Ethic Statement

This study was approved by the Ethics Committee of the CHU Sainte-Justine (ERB number 94). All the parents gave written informed consent.

### Patients and Tissue Collection

We obtained flash-frozen samples of ectopic thyroid tissue removed from 3 girls aged 8, 10 and 15 yr, because it caused local symptoms (i.e. dysphagia). For controls, we used (i) thyroid tissue from 2 girls (aged 15 and 16 yr) and 1 boy (aged 4 yr) who were operated for a single hyperfunctioning thyroid nodule that had arisen in an orthotopic thyroid and (ii) commercially available RNA from normal thyroid when appropriate (Table S5).

### Functional clusters and pathways analysis

We submitted the 1011 differentially expressed genes into gene ontology (GO) groups using the DAVID database (http://david.abcc.ncifcrf.gov) for cluster analysis according to Gene Ontology (GO) terms with medium or high classification stringency. To provide a refined analysis, the 100 validated gene were analyzed through Ingenuity Pathways Analysis (IPA; http:www.ingenuity.com), a software that also considers the level of gene expression. With either DAVID or IPA, the proportion of each gene in the submitted list is compared with the one in the whole genome to compute the P value of the Fisher's test, the enrichment scores (i.e. geometric mean of the inverse log of each P value) and the Benjamini-Hochberg multiple correction P value.

### Expression Arrays

After surgical resection, the samples were immediately frozen in liquid nitrogen and stored at −70 Celsius until use. Total RNA was extracted as per manufacturer recommendations using the QIAzol kit (QIAGEN Inc., Ontario, Canada). RNA was DNase-treated to minimize DNA contamination. RNA quantity was measured by ND-1000 (Nanodrop, Wilmington, DE, USA). RNA quality was assessed by electropherograms on the Agilent 2100 Bioanalyzer. Microarray hybridization was performed on three different ectopic thyroids (two in duplicate, one in quadruplicate) and compared to RNA of thyroid tissue from a Caucasian female, age 68 y with gall bladder cancer (Ambion, #AM6872). Double-stranded cDNA was synthesized from 1 µg of total RNA, followed by production of antisense RNA containing the modified nucleotide 5-(3-aminoallyl)-UTP using the Amino Allyl MessageAmp™ II aRNA Amplification kit (Ambion, Texas, USA). After labeling with Cy3 or Cy5 (GE Healthcare Bio-Sciences, New Jersey, USA), sample pairs were hybridized onto Human Exonic Evidence Based Oligonucleotide HEEBO slides (Stanford Functional Genomics Facility, CA, USA). The oligonucleotide set consists of 44544 70-mer probes that were designed using a transcriptome-based annotation of exonic structure for genomic loci. Hybridizations were replicated with dye swap. Slides were scanned using a Molecular Devices 4000B Laser scanner and expression levels were quantified using GenePix Pro 6.1 image analysis software (Axon Instruments, CA, USA). Image acquisitions were performed with automatic photomultiplier gains (PMT) adjustment. Artefact-associated spots were eliminated by both visual and software-guided flags, as were spots with a signal/background fluorescence ratio less than 2. The fluorescence values were imported into Acuity 4.0 software package (Molecular Devices, Union City, CA, USA). A non-linear locally weighted scatter plot (Lowess) normalization method applied to each individual block (print-tip option) was carried out using Acuity 4.0 software package (Molecular Devices, Union City, CA, USA)[28]. The identification of genes with significant differences in expression levels was performed using the significance analysis of microarray method (SAM one class) [29]. SAM estimates the percentage of genes identified by chance, the false discovery rate (FDR). We assessed the statistical significance of the differential expression of genes by computing a q-value (i.e. minimum FDR) for each gene (Table 1). Genes were considered to be differentially expressed when the absolute normalized fold change between ectopic thyroids and control was determined to be greater than 2.0 or less than 0.5 in at least one pair of the hybridized arrays. Full access to the primary array data is available on the GEO web site (http://www.ncbi.nlm.nih.gov/projects/geo/) under accession number GSE16804.

### Quantitative Real-Time RT-PCR

Validation of the expression levels of 100 genes of interest was carried out using TaqMan low density array (TLDA) technology (Applied Biosystems, Ontario, Canada). Probes and primers have been selected with the publically available software http://www5.appliedbiosystems.com/tools/ and can be retrieved by using the assay ID reported in Tables 2, 3 and S4. The expression levels were normalized to the expression level of the 18S rRNA. Induced (n = 49) and repressed (n = 51) genes were selected from the 1011 differentially regulated genes found in the HEEBO expression microarray analysis. Total RNAs from thyroids were first treated with the DNA-free kit to remove residual contamination of genomic DNA (Ambion Inc.). DNA-free total RNA (175 ng) was subjected to reverse transcription using High-Capacity cDNA Reverse Transcription Kits (Applied Biosystems). An aliquot of the cDNA was mixed to TaqMan® Gene Expression Master Mix, loaded on the TLDA plates then centrifuged for distribution of the material in the 384 wells. Gene target amplifications were performed in triplicate using the 7900HT Real-Time PCR System (Applied Biosystems). Sequence Detection System software version 2.2.2 (Applied Biosystems) was used for comparative gene expression analysis using the ÄCt method. In a first analysis, expression levels found in the normal orthotopic thyroid from Ambion were compared to the mRNA levels present in the ectopic thyroids. Expression levels in the hot nodules were then compared to the levels found in the ectopic thyroids. For analysis, the cut-off log_2_ value was 0.5. Then, to compare the results of the quantitative real-time RT-PCR with those of expression arrays, Pearson correlation was calculated with the free statistical software R [30].

### Methylation Profiling by Methylated DNA Immunoprecipitation (MeDIP) and MeDIP-chip

The MeDIP-chip was performed using pairs of enriched methylated fraction (IP) and normal fraction (IN) of genomic DNA from our three ectopic thyroids and three controls (i.e. hyperfunctioning thyroid nodules). The methylated fraction of genomic DNA was enriched using the methylated DNA immunoprecipitation (MeDIP) assay [31] and interrogated on human Promoter plus CpG Island Tiling Arrays with a ChIP design for CpG islands and promoter regions (n = 28,226) from HG18 using 385,020 Probes selected from CGH probe bank with a median spacing of 101bp (Roche NimbleGen, Madison, WI). Briefly, 4 µg *Mse*I digested genomic DNA was immunoprecipitated with monoclonal mouse anti 5-methylcytidine antibody (New England Biolab, Pickering, Ontario and Abcam Inc.Cambridge, MA 02139). After washes and purification steps, immunoprecipitated material and a sample of input DNA were amplified using GenomePlex Complete Whole Genome Amplification (WGA) kit (Sigma-Aldrich, Saint Louis, Missouri 63103 USA). The resulting products (4 µg) were labeled, cohybridized and scanned by the NimbleGen Customer Service (Roche NimbleGen, Madison, WI). For each sample, NimbleScan detects peaks by searching for at least two probes above a P-value cutoff (−log_10_) of 2 and peaks within 500bp are merged (gff files on GEO web site). Then, peak data were analyzed to compare the methylation profile between ectopic thyroids and orthotopic thyroids using the Loess normalized log_2_ (ChIP/input) ratios with the one-way ANOVA tool of the Partek Genomics Suite (PGS) software. A FDR less than 0.1 was considered as significant. Full access to the primary array data is available on the GEO web site (http://www.ncbi.nlm.nih.gov/projects/geo/) under accession number GSE17581.

### Array Comparative Genomic Hybridization and validation with quantitative real-time PCR

Array comparative genomic hybridization (aCGH) was performed using pairs of thyroid tissues and leucocytes from the three ectopic thyroids. We used the Nimblegen X1 HG18 whole genome CGH design (version 2). The 385,815 probes are distributed across the genome with a median spacing of 7073 bp. Probes are 60-mers, with a Tm target of 80 degrees. Labeling, hybridization, washing and scanning was performed by the NimbleGen Customer Service (Roche NimbleGen, Madison, WI). After normalization, the log_2_ (test/reference) signals were analyzed using a circular binary segmentation algorithm (segMNT) with the PGS Software to identify somatically acquired segmental copy number changes. To call a copy number change, segMNT required a segment to span a minimum of 5 consecutive probes with a p-value threshold of 0.001 and a signal to noise ratio of 0.3. Then, reported regions were set at segMNT log_2_ ratio below 0.3 or above 0.2 in all three samples with a p-value threshold of 0.01. Full access to the primary array data is available on the GEO web site (http://www.ncbi.nlm.nih.gov/projects/geo/) under accession number GSE17463. Validation of the aCGH with quantitative real-time PCR was performed with the TaqMan technology. Identified CNVs were validated using TaqMan Gene Copy Number Assays from ABI. Probes and primers have been selected with the public available software (http://www5.appliedbiosystems.com/tools/cnv/) and can be retrieved by using the assay ID reported in Table S5.

## Supporting Information

Figure S1 Reliability of the HEEBO expression array was confirmed by calculating the Pearson correlation coefficient (r = 0,86; P<2.2 e-16, n = 100 genes; ectopic thyroid (n = 3) vs normal thyroid - Ambion, #AM6872) between microarray and qRT-PCR results. Results are expresed in log2 ratio.
(0.01 MB PDF)

Table S1 The top three clusters for the 1011 differentially expressed genes (more than two-fold induced or repressed).
(1.92 MB TIF)

Table S2 Clusters for the induced (n = 522) and repressed (n = 489) genes.
(0.09 MB PDF)

Table S3 Source of patients derived thyroid tissues.
(0.02 MB PDF)

Table S4 Validated genes (n = 81) with divergent expression in ectopic thyroid tissue (i.e. dependent on the activation state and independent of the localization of the thyroid tissue).
(0.07 MB PDF)

Table S5 Thyroid specific CNVs found in ectopic tissues.
(0.05 MB PDF)
