# Supplementary figures and images for: Transcriptome, Methylome and Genomic Variations Analysis of Ectopic Thyroid Glands
Source: PLoS One. 2010 Oct 15;5(10):e13420. doi: 10.1371/journal.pone.0013420 (PMC2955549; doi:10.1371/journal.pone.0013420)

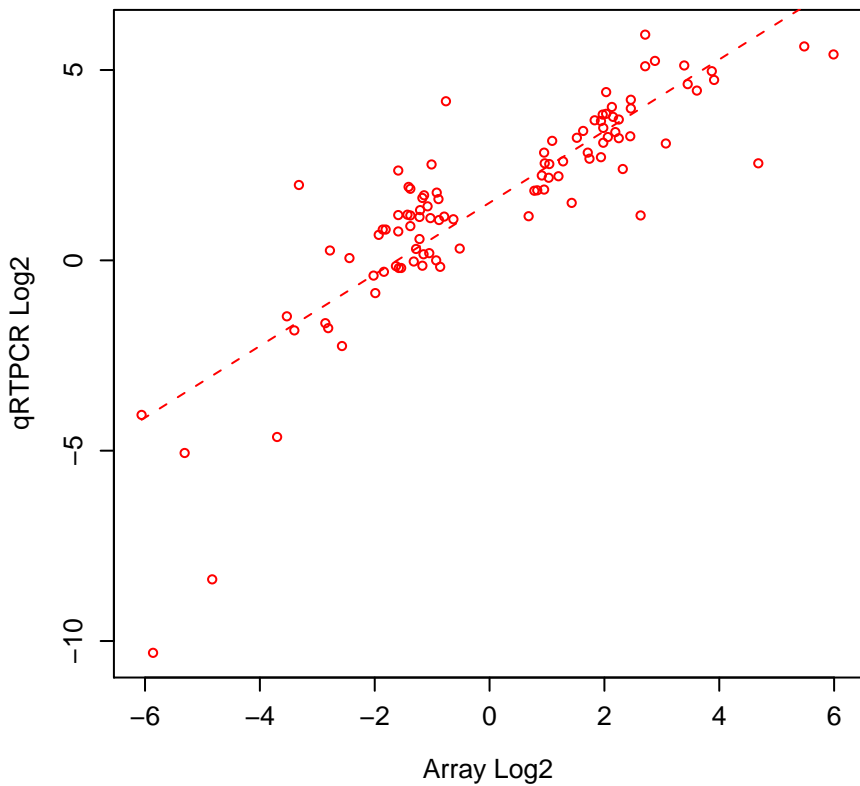

Supplement: Figure S1 — Reliability of the HEEBO expression array was confirmed by calculating the Pearson correlation coefficient (r = 0,86; P<2.2 e-16, n = 100 genes; ectopic thyroid (n = 3) vs normal thyroid - Ambion, #AM6872) between microarray and qRT-PCR results. Results are expresed in log2 ratio. (0.01 MB PDF) [file pone.0013420.s001.pdf]

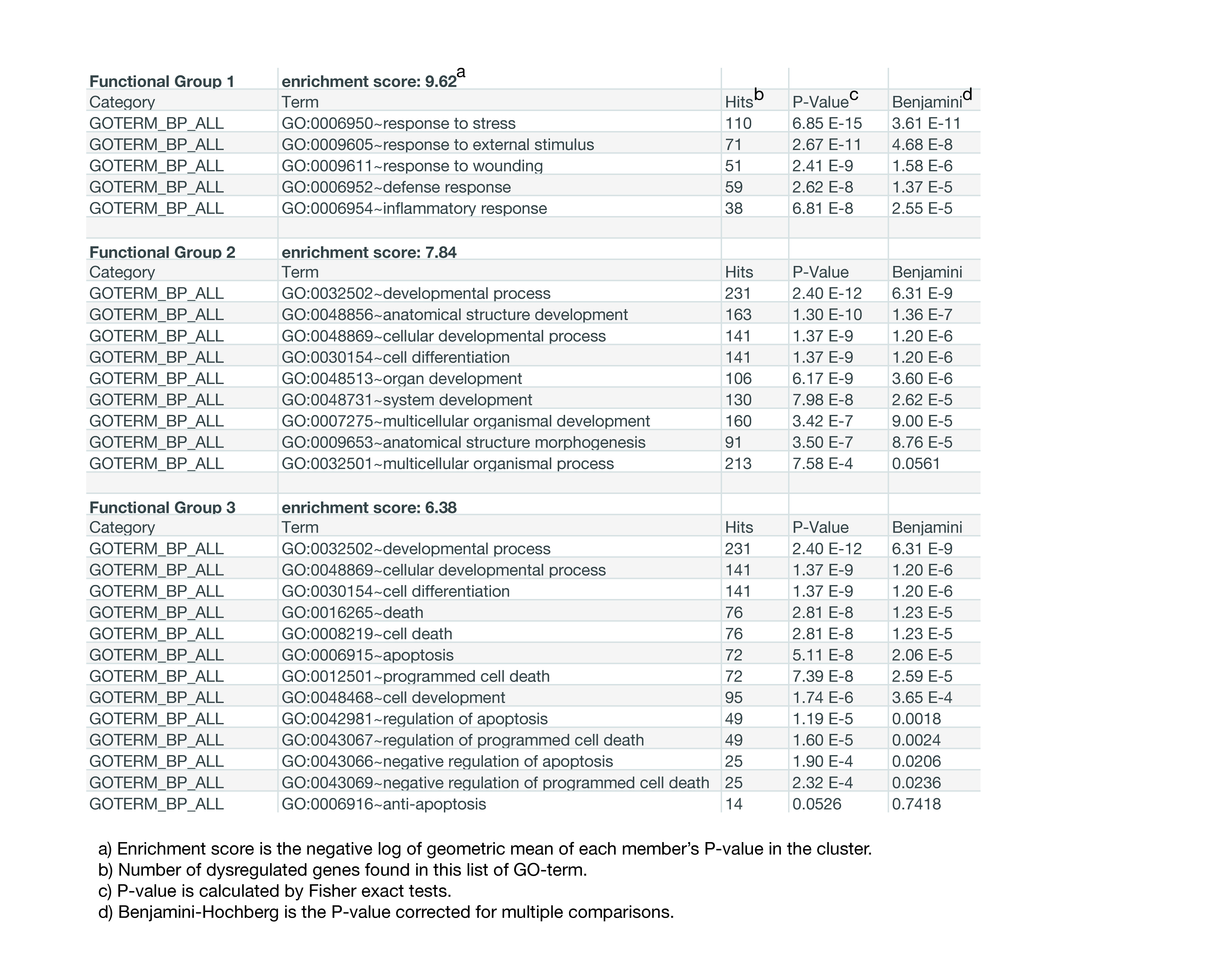

Supplement: Table S1 — The top three clusters for the 1011 differentially expressed genes (more than two-fold induced or repressed). (1.92 MB TIF) [file pone.0013420.s002.tif]
