# Supplementary material for: Transcriptome, Methylome and Genomic Variations Analysis of Ectopic Thyroid Glands
Source: PLoS One. 2010 Oct 15;5(10):e13420. doi: 10.1371/journal.pone.0013420 (PMC2955549; doi:10.1371/journal.pone.0013420)
Supplement: Table S2 — Clusters for the induced (n = 522) and repressed (n = 489) genes. (0.09 MB PDF) [file pone.0013420.s003.pdf]

**induced genes**				
Functional Group 1	Enrichment score (a): 10.44			
Category	Term	Hits	PValue (b)	Benjamini (c)
GOTERM_BP_ALL	GO:0048856~anatomical structure development	107	7.91 E-13	4.15 E-9
GOTERM_BP_ALL	GO:0048731~system development	88	1.48 E-10	1.94 E-7
GOTERM_BP_ALL	GO:0007275~multicellular organismal development	106	3.83 E-10	3.36 E-7
Functional Group 2	Enrichment score: 7.06			
Category	Term	Hits	PValue	Benjamini
GOTERM_BP_ALL	GO:0001568~blood vessel development	22	8.90 E-9	5.84 E-6
GOTERM_BP_ALL	GO:0001944~vasculature development	22	1.18 E-8	6.21 E-6
GOTERM_BP_ALL	GO:0048514~blood vessel morphogenesis	19	1.63 E-7	5.04 E-5
GOTERM_BP_ALL	GO:0048646~anatomical structure formation	19	5.08 E-7	1.48 E-4
GOTERM_BP_ALL	GO:0001525~angiogenesis	17	5.21 E-7	1.44 E-4
Functional Group 3	Enrichment score: 6.79			
Category	Term	Hits	PValue	Benjamini
SP_PIR_KEYWORDS	extracellular matrix	23	1.39 E-8	2.96 E-6
GOTERM_CC_ALL	GO:0005578~proteinaceous extracellular matrix	27	4.61 E-7	1.33 E-4
GOTERM_CC_ALL	GO:0031012~extracellular matrix	27	6.44 E-7	1.39 E-4
Functional Group 4	Enrichment score: 5.84			
Category	Term	Hits	PValue	Benjamini
GOTERM_BP_ALL	GO:0048534~hemopoietic or lymphoid organ	20	9.35 E-7	2.45 E-4
GOTERM_BP_ALL	GO:0030097~hemopoiesis	19	1.42 E-6	3.41 E-4
GOTERM_BP_ALL	GO:0002520~immune system development	20	2.19 E-6	5.01 E-4
Functional Group 5	Enrichment score: 5.76			
Category	Term	Hits	PValue	Benjamini
SP_PIR_KEYWORDS	triple helix	11	2.84 E-9	1.01 E-6
SP_PIR_KEYWORDS	hydroxylysine	10	6.70 E-8	8.92 E-6
SP_PIR_KEYWORDS	hydroxyproline	10	1.15 E-7	1.36 E-5
INTERPRO	IPR008160:Collagen triple helix repeat	13	4.68 E-7	0.0027
INTERPRO	IPR008161:Collagen helix repeat	11	6.05 E-7	0.0017
SP_PIR_KEYWORDS	hydroxylation	11	2.08 E-6	1.71 E-4
GOTERM_BP_ALL	GO:0006817~phosphate transport	13	3.49 E-6	6.54 E-4
SP_PIR_KEYWORDS	collagen	12	5.54 E-5	0.0036
GOTERM_BP_ALL	GO:0015698~inorganic anion transport	14	2.02 E-4	0.0276
GOTERM_BP_ALL	GO:0006820~anion transport	15	4.35 E-4	0.0570
**repressed genes**				
Functional Group 1	Enrichment score: 6.24			
Category	Term	Hits	PValue	Benjamini
SMART	SM00427:H2B	7	1.65 E-7	1.00 E-4
INTERPRO	IPR000558:Histone H2B	7	1.95 E-7	5.79 E-4
PIR_SUPERFAMILY	PIRSF002050:histone H2B	7	4.72 E-7	0.001
INTERPRO	IPR009072:Histone-fold	9	6.66 E-6	0.013
Functional Group 2	Enrichment score: 5.24			
Category	Term	Hits	PValue	Benjamini
GOTERM_BP_ALL	GO:0006915~apoptosis	40	1.30 E-6	7.60 E-4
GOTERM_BP_ALL	GO:0012501~programmed cell death	40	1.62 E-6	8.55 E-4
GOTERM_BP_ALL	GO:0016265~death	41	2.34 E-6	9.47 E-4
GOTERM_BP_ALL	GO:0008219~cell death	41	2.34 E-6	9.47 E-4
GOTERM_BP_ALL	GO:0048468~cell development	46	5.35 E-4	0.086
Functional Group 3	Enrichment score: 5.14			
Category	Term	Hits	PValue	Benjamini
INTERPRO	IPR007125:Histone core	12	3.44 E-10	2.03 E-6
SP_PIR_KEYWORDS	Nucleosome core	12	1.38 E-9	7.38 E-7
GOTERM_BP_ALL	GO:0006334~nucleosome assembly	13	5.01 E-7	4.39 E-4
GOTERM_CC_ALL	GO:0000786~nucleosome	12	5.87 E-7	1.02 E-4
GOTERM_BP_ALL	GO:0031497~chromatin assembly	13	2.21 E-6	0.0010
INTERPRO	IPR009072:Histone-fold	9	6.66 E-6	0.0130
GOTERM_BP_ALL	GO:0006333~chromatin assembly or disassembly	14	1.74 E-5	0.0057
GOTERM_BP_ALL	GO:0065004~protein-DNA complex assembly	14	3.30 E-5	0.0086
SP_PIR_KEYWORDS	Chromosomal protein	12	7.27 E-5	0.0077
GOTERM_CC_ALL	GO:0000785~chromatin	14	4.76 E-4	0.0194
GOTERM_BP_ALL	GO:0006323~DNA packaging	18	7.10 E-4	0.0936
GOTERM_BP_ALL	GO:0006325~establishment and/or maintenance of	17	0.0015	0.1809
GOTERM_BP_ALL	GO:0051276~chromosome organization and	19	0.0026	0.2339
Functional Group 4	Enrichment score: 4.51			
Category	Term	Hits	PValue	Benjamini
GOTERM_CC_ALL	GO:0043229~intracellular organelle	228	1.09 E-5	0.0010
GOTERM_CC_ALL	GO:0043226~organelle	228	1.14 E-5	9.91 E-4
GOTERM_CC_ALL	GO:0043231~intracellular membrane-bound	199	8.33 E-5	0.0055
GOTERM_CC_ALL	GO:0043227~membrane-bound organelle	199	8.58 E-5	0.0053
Functional Group 5	Enrichment score: 4.33			
Category	Term	Hits	PValue	Benjamini
GOTERM_CC_ALL	GO:0043292~contractile fiber	10	2.36 E-5	0.0018
GOTERM_CC_ALL	GO:0030016~myofibril	9	4.46 E-5	0.0032
GOTERM_CC_ALL	GO:0044449~contractile fiber part	9	9.17 6E-5	0.0052

[a]Enrichment score is the negative log of geometric mean of each member's P-value in the cluster.
[b]P-value is calculated by Fisher's exact tests.
[c]Benjamini-Hochberg is the P-value corrected for multiple comparisons.
