# Supplementary material for: Transcriptome, Methylome and Genomic Variations Analysis of Ectopic Thyroid Glands
Source: PLoS One. 2010 Oct 15;5(10):e13420. doi: 10.1371/journal.pone.0013420 (PMC2955549; doi:10.1371/journal.pone.0013420)
Supplement: Table S3 — Source of patients derived thyroid tissues. (0.02 MB PDF) [file pone.0013420.s004.pdf]

	case 1	case 2	case 3	control 1	control 2	control 3	control AMBION
**thyroid gland location**	ectopic - lingual	ectopic - sublingual	ectopic - sublingual	orthotopic	orthotopic	orthotopic	orthtotopic
**age at diagnosis / sexe**	8.5 yr/F	2 d/F	2 d/F	4/M	15/F	16/F	68 / F
**TSH (mU/L) prior to L-T4 treatment**	6.6	N/A	N/A	N/A	N/A	N/A	N/A
**fT4 (pmol/L) prior to L-T4 treatment**	8.5	N/A	N/A	N/A	N/A	N/A	N/A
**LT4 daily dose (µg)**	75	68.5	50	N/A	N/A	N/A	N/A
**age at surgery (yr)**	9.5	9	14	4	15	16	68
**TSH (mU/L) prior to surgery**	0.08	3.9	3.4	0.08	<0.03	0.01	N/A
**fT4 (pmol/L) prior to surgery**	15.7	14.0	12.8	24.8	22.07	17.3	N/A
