# Supplementary material for: Transcriptome, Methylome and Genomic Variations Analysis of Ectopic Thyroid Glands
Source: PLoS One. 2010 Oct 15;5(10):e13420. doi: 10.1371/journal.pone.0013420 (PMC2955549; doi:10.1371/journal.pone.0013420)
Supplement: Table S4 — Validated genes (n = 81) with divergent expression in ectopic thyroid tissue (i.e. dependent on the activation state and independent of the localization of the thyroid tissue). (0.07 MB PDF) [file pone.0013420.s005.pdf]

Symbol	Gene product	Enterz Gene	mean log2 ratio on HEEBO array (3 ectopic thyroids VS control AMBION)	RQ of qRTPCR vs normal thyroid (log2 converted)	RQ of qRTPCR vs hot nodules (log2 converted)	Taq Man Assay ID
**CHGA**	chromogranin A (parathyroid secretory protein 1)	1113	-4,83	-8,38	5,15	Hs00900373_m1
**TNNC2**	troponin C2, fast	7125	-3,7	-4,64	4,67	Hs00268519_m1
**ACTA1**	actin, alpha 1, skeletal muscle	58	-5,31	-5,06	2,88	Hs00559403_m1
**THRSP**	thyroid hormone responsive (SPOT14 homolog, rat)	7069	-1,01	2,52	1,97	Hs00930058_m1
**GNG5**	guanine nucleotide binding protein (G protein), gamma 5	2787	-1,22	0,56	1,75	Hs00359134_g1
**SMOC2**	SPARC related modular calcium binding 2	64094	-1,32	-0,03	1,49	Hs00405777_m1
**TEF**	thyrotrophic embryonic factor	7008	-1,17	-0,13	1,21	Hs00162657_m1
**CNTN6**	contactin 6	27255	-2,86	-1,64	1,15	Hs00274291_m1
**LRP8**	low density lipoprotein receptor-related protein 8, apolipoprotein e receptor	7804	-1,38	1,88	1,09	Hs00182998_m1
**EDN3**	endothelin 3	1908	-3,4	-1,84	0,99	Hs00171177_m1
**FMR1**	fragile X mental retardation 1	2332	-1,08	1,42	0,92	Hs00924547_m1
**GJA1**	gap junction protein, alpha 1, 43kDa (connexin 43)	2697	-1,59	2,36	0,92	Hs00748445_s1
**CKM**	creatine kinase, muscle	1158	-5,86	-10,31	0,74	Hs00176490_m1
**SPRED1**	sprouty-related, EVH1 domain containing 1	161742	-1,05	0,19	0,71	Hs00544790_m1
**CHORDC1**	cysteine and histidine-rich domain (CHORD)-containing, zinc binding protein 1	26973	-1,93	0,67	0,62	Hs00854389_g1
**PLEKHA3**	pleckstrin homology domain containing, family A (phosphoinositide binding specific) member 3	65977	-1,21	1,32	0,57	Hs00604551_m1
**MTHFD2**	methylenetetrahydrofolate dehydrogenase (NADP+ dependent) 2, methenyltetrahydrofolate cyclohydrolase	10797	-1,86	0,81	0,48	Hs00759197_s1
**SMAD9**	SMAD, mothers against DPP homolog 9 (Drosophila)	4093	-0,52	0,31	0,42	Hs00195441_m1
**SMAD5**	SMAD, mothers against DPP homolog 5 (Drosophila)	4090	-0,79	1,15	0,42	Hs00195437_m1
**TNFRSF21**	tumor necrosis factor receptor superfamily, member 21	27242	1,98	3,48	0,4	Hs00205419_m1
**MUC1**	mucin 1, transmembrane	4582	-1,63	-0,1	0,39	Hs00159357_m1
**PRKCE**	protein kinase C, epsilon	5581	-3,32	1,98	0,38	Hs00178455_m1
**EMP3**	epithelial membrane protein 3	2014	2,06	3,24	0,38	Hs00171319_m1
**INHBB**	inhibin, beta B (activin AB beta polypeptide)	3625	-2,02	-0,4	0,35	Hs00173582_m1
**EVC2**	Ellis van Creveld syndrome 2 (limbin)	132884	-1,43	1,19	0,34	Hs00377633_m1
**ARHGEF6**	Rac/Cdc42 guanine nucleotide exchange factor (GEF) 6	9459	2,25	3,21	0,31	Hs00374462_m1
**FOS**	v-fos FBJ murine osteosarcoma viral oncogene homolog	2353	3,61	4,46	0,26	Hs99999140_m1
**TSHR**	thyroid stimulating hormone receptor	7253	1,43	1,49	0,2	Hs01053846_m1
**POLD4**	polymerase (DNA-directed), delta 4	57804	0,95	1,86	0,2	Hs00221775_m1
**RASD1**	RAS, dexamethasone-induced 1	51655	-3,53	-1,47	0,12	Hs00607394_g1
**EGFR**	epidermal growth factor receptor (erythroblastic leukemia viral (v-erb-b) oncogene homolog, avian)	1956	-0,88	1,06	0,08	Hs00193306_m1
**PKNOX1**	PBX/knotted 1 homeobox 1	5316	-1,59	1,19	0,07	Hs00231814_m1
**TCF4**	transcription factor 4	6925	0,78	1,83	0,04	Hs00162613_m1
**NAV1**	neuron navigator 1	89796	1,74	2,67	0,01	Hs00368110_m1
**FZD4**	frizzled homolog 4 (Drosophila)	8322	0,91	2,23	0,01	Hs00201853_m1
**PLXND1**	plexin D1	23129	1,97	3,83	0	Hs00391129_m1
**ACP2**	acid phosphatase 2, lysosomal	53	1,04	2,53	0	Hs00155636_m1
**SFRS2**	splicing factor, arginine/serine-rich 2	6427	2,03	4,42	-0,01	Hs00293258_m1
**DTX4**	deltex 4 homolog (Drosophila)	23220	-1,84	-0,3	-0,02	Hs00392288_m1
**CTNNAL1**	catenin (cadherin-associated protein), alpha-like 1	8727	-1,59	0,76	-0,03	Hs00169384_m1
**PAX8**	paired box gene 8	7849	4,68	2,55	-0,04	Hs00243335_m1
**CXCL12**	chemokine (C-X-C motif) ligand 12 (stromal cell-derived factor 1)	6387	1,94	2,71	-0,04	Hs00930455_m1
**SFRP1**	secreted frizzled-related protein 1	6422	2,88	5,24	-0,07	Hs00610060_m1
**TG**	thyroglobulin	7038	1,2	2,21	-0,07	Hs00174974_m1
**EFNB2**	ephrin-B2	1948	0,95	2,83	-0,07	Hs00187950_m1
**ROBO4**	roundabout homolog 4, magic roundabout (Drosophila)	54538	1,63	3,4	-0,1	Hs00219408_m1
**ASPM**	asp (abnormal spindle)-like, microcephaly associated (Drosophila)	259266	-0,76	4,18	-0,12	Hs00396967_m1
**FN1**	fibronectin 1	2335	1,94	3,66	-0,13	Hs00415006_m1
**AKT3**	v-akt murine thymoma viral oncogene homolog 3 (protein kinase B, gamma)	10000	-1,14	1,71	-0,13	Hs00289302_s1
**CYBRD1**	cytochrome b reductase 1	79901	-1,17	1,63	-0,13	Hs00227411_m1
**CXCR4**	chemokine (C-X-C motif) receptor 4	7852	0,68	1,16	-0,15	Hs00237052_m1
**FZD3**	frizzled homolog 3 (Drosophila)	7976	-0,63	1,08	-0,15	Hs00184043_m1
**AKT1**	v-akt murine thymoma viral oncogene homolog 1	207	-0,89	1,8	-0,18	Hs00920503_m1
**LMO3**	LIM domain only 3 (rhombotin-like 2)	55885	-1,15	0,16	-0,18	Hs00375237_m1
**NLK**	nemo like kinase	51701	1,03	2,17	-0,18	Hs00212076_m1
**DKK3**	dickkopf homolog 3 (Xenopus laevis)	27122	2,15	3,77	-0,2	Hs00247429_m1
**LRP8**	low density lipoprotein receptor-related protein 8, apolipoprotein e receptor	7804	-1,38	0,9	-0,2	Hs00182998_m1
**CTGF**	connective tissue growth factor	1490	2,45	3,26	-0,22	Hs00170014_m1
**HSPA1B**	heat shock 70kDa protein 1B	3304	-2,57	-2,25	-0,22	Hs00359147_s1
**FGFR1**	fibroblast growth factor receptor 1 (fms-related tyrosine kinase 2, Pfeiffer syndrome)	2260	2,32	2,4	-0,23	Hs00241111_m1
**KPNA4**	karyopherin alpha 4 (importin alpha 3)	3840	-1,22	1,14	-0,25	Hs00927639_g1
**SNX1**	sorting nexin 1	6642	-1,38	1,17	-0,25	Hs00541723_m1
**VEGFA**	vascular endothelial growth factor	7422	3,07	3,07	-0,27	Hs00900055_m1
**FZD1**	frizzled homolog 1 (Drosophila)	8321	-1,28	0,3	-0,27	Hs00268943_s1
**LAMA4**	laminin, alpha 4	3910	2,13	4,03	-0,29	Hs00158588_m1
**CDC42EP4**	CDC42 effector protein (Rho GTPase binding) 4	23580	-1,54	-0,2	-0,29	Hs00201664_m1
**CEBPB**	CCAAT/enhancer binding protein (C/EBP), beta	1051	-1,58	-0,2	-0,37	Hs00270923_s1
**TPO**	thyroid peroxidase	7173	-0,93	0	-0,43	Hs00174927_m1
**DMD**	dystrophin (muscular dystrophy, Duchenne and Becker types)	1756	-1,41	1,93	-0,49	Hs01049436_m1
**FXR1**	fragile X mental retardation, autosomal homolog 1	8087	-1,03	1,11	-0,64	Hs01096865_m1
**DUOX2**	dual oxidase 2	50506	2,46	3,99	-0,66	Hs00204187_m1
**CCND1**	cyclin D1 (PRAD1: parathyroid adenomatosis 1)	595	0,96	2,55	-0,86	Hs00277039_m1
**DIO1**	deiodinase, iodothyronine, type I	1733	2,71	5,1	-0,89	Hs00174944_m1
**DIO2**	deiodinase, iodothyronine, type II	1734	2,03	3,85	-0,89	Hs00255341_m1
**CLDN5**	claudin 5 (transmembrane protein deleted in velocardiofacial syndrome)	7122	-2,44	0,06	-0,92	Hs01561351_m1
**FGF12**	fibroblast growth factor 12	2257	2,19	3,37	-1,03	Hs00374427_m1
**CDH16**	cadherin 16, KSP-cadherin	1014	-1,81	0,81	-1,43	Hs00187880_m1
**CDH2**	cadherin 2, type 1, N-cadherin (neuronal)	1000	-0,86	-0,16	-2,0	Hs00169953_m1
**FOSB**	FBJ murine osteosarcoma viral oncogene homolog B	2354	5,48	5,61	-2,73	Hs00171851_m1
**PAX2**	paired box gene 2	5076	-0,92	1,78	-3,47	Hs01057416_m1
**MYL2**	myosin, light polypeptide 2, regulatory, cardiac, slow	4633	-2,78	0,26	-4,32	Hs00166405_m1
