# Supplementary material for: Transcriptome, Methylome and Genomic Variations Analysis of Ectopic Thyroid Glands
Source: PLoS One. 2010 Oct 15;5(10):e13420. doi: 10.1371/journal.pone.0013420 (PMC2955549; doi:10.1371/journal.pone.0013420)
Supplement: Table S5 — Thyroid specific CNVs found in ectopic tissues. (0.05 MB PDF) [file pone.0013420.s006.pdf]

chromosome	start	end	length (bps)	classification	genes (Entrez Gene ID)	Validation with qPCR: case (number of copies)	Assay ID		
**1p36.32**	3419310	3504625	85316	common CNV	MEGF6 (1953)	case 1 (3)	Hs02299905_cn	Hs03346798_cn	Hs03344891_cn
**4p16.3**	1696020	1781125	85106	common CNV	FGFR3 (2261); TACC3 (10460)	case 1 (4); case 3 (3)	Hs00091802_cn	Hs03518017_cn	
**7q11.23**	74616550	74711750	95201	common CNV	LOC750805 similar to PMS2L14 (750805); WBSCR16 (81554); GATSL2(729438)	ø	*	*	*
**12q24.33**	131582000	131678000	96001	common CNV	AUTS2L (304419); FBRSL1(57666)	case 1 (3); case 3 (3)	Hs03817872_cn	Hs03816047_cn	Hs03835002_cn

*Custom made qPCR for 7q11.23 are described in the table below.

Assay ID	Forward Primer Seq.	Reverse Primer Seq.	Probe
chr7_CCWRDIS	ATGTCCCCCTACTCAGTTCTCA	CTCCGAGGTCCCCAAAGATTG	CTCCGAGGTCCCCAAAGATTG
ch7-2_CCY89U8	GGCCCGGACCCTTCCTA	GTACCAGCTCATGGCAGTCT	GTACCAGCTCATGGCAGTCT
ch7-3_CC7ZW2S	GCACCAGCCTGGAAGGA	CAAACTCCCTCATTCCAGACAGAA	CAAACTCCCTCATTCCAGACAGAA
